# Differential Attachment of Pulmonary Cells on PDMS Substrate with Varied Features

**DOI:** 10.22037/ijpr.2020.112214.13608

**Published:** 2020

**Authors:** Mojdeh Mohseni, Sahar Barzegari Banadkoki, Abolfazl Dashti, Golrokh Farnam, Kamyar Keshavarz F., Farshad H. Shirazi

**Affiliations:** a *Radiation Biology Research Center, Iran University of Medical Sciences, Tehran, Iran. *; b *Pharmaceutical Sciences Research Center, Shahid Beheshti University of Medical Sciences, Tehran, Iran. *; c *Department of Toxicology, School of Pharmacy, Shahid Beheshti University of Medical Sciences, Tehran, Iran.*

**Keywords:** Roughness, Stiffness, Polymeric substrate, Cell attachment, Lung cancer

## Abstract

Cancer is now a global concern, and control of the function of cancer cells is recognized as an important challenge. Although many aggressive chemical and radiation methods are in practice to eliminate cancer cells, most of them imply severe adverse toxic effects on patients. Taking advantage of natural physical differences between cancer and normal cells might benefit the patient with more specific cytotoxicity and fewer adverse effects. Physical factors are the main means that can influence cell-biomaterial interaction. To explore the importance of attachment phenomena on cancer cells in this research, polydimethylsiloxane (PDMS) substrates with varied stiffness and roughness were synthesized and lung cancer cell^’^s behavior on these surfaces was examined. To achieve diverse surface topography SDBD plasma was used at various exposure times, and different stiffness was obtained by changing in curing agent amount. Atomic force microscopy (AFM) and tensile modulus were employed to the characterization of roughness and stiffness respectively. Lung cancer cell survival and growth were studied by MTT and image processing analysis. The results indicated that softer and rougher surface made lung cancer cells to die. The number of detached cells, mean space of the detached cells, cellular coverage of surface, and the ratio of detached/ all cellular coverage were significantly affected by roughness and stiffness. Therefore, physical factors can control cell function, especially in lung cancer cells and these results might provide a strong base to help cancer cell removal.

## Introduction

 Recent scientific efforts in regenerative medicine as a promising approach for the treatment of various diseases have focused on surface substitute engineering (1). In this insight, cell response in contact with biomaterials can be modulated by surface features (2). Surface chemistry, topography, and mechanical stiffness are some means which can direct cellular reaction toward a specific response to control the fate and function of cells (3). As changing in microenvironment cell properties can alter cell signaling pathways, and human cancer cell lines are responsive to variations in microenvironmental stiffness and roughness is unclear, an appropriate surface design to obtain desirable biological function can help us to obtain excellent models for particular cellular behavior (4, 5)^.^

Among different cell functions, adhesion plays an important role in cell communication and regulation (6). Alterations in cell adhesion have been seen in different diseases, and also reduced cell adhesion in cancer cells can promote to decline cell signaling. Hence, adjustment of cell adhesion may regulate cell behavior in many biological disorders (7). 

A wide range of biomaterials especially polymers have been utilized for cell imprinting and interface interaction study. Polyacrylamide and hyaluronan derivatives are some examples of polymers that have been used for this purpose. Unique features such as transparency, ease of handling and ability to make desired micro and nanostructure in polydimethylsiloxane (PDMS) made this synthetic polymer as the best candidate for cell interaction evaluation studies (8).

To our knowledge, attachment and morphological features of A549 as a model of adenocarcinoma human alveolar basal epithelial cells in contact with surface rigidity and roughness has not yet been studied. A549 cell line is an example of very resistant tumor cells to therapy. Hence, in this study, several PDMS substrates with varying roughness and stiffness are prepared to further evaluate the A549 cells attachment, proliferation and morphological characteristics on these surfaces. Results have been processed using MATLAB software for better interpretation of surface stiffness and roughness characteristics on cell morphology and survival. These findings might support new means to treat cancer cells by varying the *in-situ* environment.

## Experimental

*Materials*

SYLGARD 184 (silicone elastomer kit) was purchased from Dow Corning, Tokyo, Japan. DMEM Medium, fetal bovine serum (FBS), penicillin-streptomycin (10,000 U/mL), and trypsin (0.25%) were supplied from GIBCO (USA). 3-(4, 5-Dimethylthiazol-2-yl)-2,5-diphenyltetrazolium bromide (MTT) and dimethyl sulfoxide (DMSO) were purchased from Merck (Germany). 

*PDMS fabrication*

Three compositions of precursor and curing agent were mixed and the samples of S1, S2, and S3 with the ratio of 2.5, 5, and 10 bases were prepared, respectively. In the procedure of PDMS fabrication, following the mechanical stirring each sample was degassed under vacuum for 20 min and the mixture was poured into the polystyrene mold and cured at 75 °C for 24 h. The circular shape of platforms with the dimension of 20 × 4 mm was taken for atomic force microscopy analysis and cell culture evaluations. 

*Surface plasma treatment*

To gain assorted roughness in PDMS substrates, SDBD plasma (Iran, Shahid Beheshti University) was applied. The proportion of base to curing agent for plasma treatment was set at 10:1 and the plasma power and electrode distances were 30 W and 2 mm, respectively. Different roughness in the PDMS surface was accomplished by different processing durations of 30, 90, and 180 s (R2, R3, and R4). The samples without any plasma exposure were selected as control (R1). 

*Stiffness evaluation*

 The bulk elasticity was measured by the tensile test (INSTRON 5566, United State). The substrates were cut into 30 × 6 mm in a rectangular shape and the test rate was set at 0.1 mm s^-1^. 

*Roughness characterization*

 Surface topography was analyzed by atomic force microscopy (AFM) (Nano wizard II, Germany) using none- contact (tapping) mode in the air and a frequency of 113 kHz. Each exposure time was repeated for three samples and the average of quantitative root mean square roughness was reported. 

*Cell Survival Assay*

 A549 adenocarcinoma human alveolar basal epithelial cells (1 × 10^4^ cells) were grown in DMEM medium, supplemented with 10% (v/v) heat-inactivated fetal bovine serum, and 1% (v/v) = penicillin and streptomycin. The cells were maintained at 37 °C in a humidified atmosphere containing 5% CO_2_ for 22 h. A ratio of adhered live cells was calculated by MTT assay. In this assay, the yellow tetrazolium salt (MTT) is reduced to the purple formazan dye by mitochondrial dehydrogenases in live cells. Briefly, the culture medium was replaced with fresh medium containing MTT solution (final concentration 0.5 mg/mL) and incubated for 4 h at 37 °C. Then the medium was removed and 500 μL DMSO was added to each well and mixed properly until blue formazan product completely dissolved. Absorbance was measured at the wavelength of 570 against 630 nm as the reference wavelength in an automated plate reader (BIOTEK).

*Calculation of Attached Cells Surface on PDMS*

 The image processing method has been used to calculate the percentage of PDMS surface coverage by A549 cells. To do so, the cell images of previously selected locations of PDMS growing surface were recorded at the same sizes of RGB in the values of 1200x1600x3 in jpg format. Computation MATLAB 2014b software (MATHWIRKS, Natick, MA, USA) has been used to run a subroutine on each picture.

Image enhancement (sharpening, deblurring, highlighting edges, improving image contrast, and removing noise) has been used to extract suitable features for a better result. 

Lens effects on picture quality have been erased using the average color substitution method applied to the original picture. Next, RGB images or color maps conversion have been used to produce a grayscale image. A morphological structuring element has been created to identify background surfaces. Subtraction of the resulted image from the grayscale image has been performed for better discrimination of the cells from the background. Then, image adjustment has been applied to the resulted cell image using the proper mask filter. Afterward, the resulted grayscale image was converted to binary and filtered again to reduce noise and provide better recognition of the cells’ population. Using an appropriate threshold on the resulted picture, the number of white pixels was counted versus black pixels as the representation of the surface ratio was covered by the cells in that selected area of photography. Equation 1 has been used to calculate the percentages of cellular coverage on PDMS for any particular experiment:

Number of white pixels of Image Number of rows of Image ×Number of columns of Image ×100

 (Equation 1) 

The denominator of Equation 1 results in the number of all pixels in the selected Image. This equation is used for the calculation of Figure 5. 

*Discrimination of detached cells and contribution of their cellular coverage*

With the assumption that the attached cells are not circular and the detached ones are more likely to be in circle form, computation MATLAB 2014b software (MATHWORKS, Natick, MA, USA) has been used to identify the circularity of the cells’ picture using circular Hough transformation in the specified radius range and sensitivity. As a result, the circles were colored green and the cells being blue on the black background of the cell growth media. The software has then scanned all the pixels in different colors as the representation of total detached (circled in green) surface area versus total attached (colored in blue) cells surface area using Equation 2, which is used for the process of Figure 6. 

Number of green pixels in Image Number of green pixels +Number of blue pixels in Image ×100

(Equation 2)

To consider and correct the results of the above equation for the variable sizes of circular detached cells, Equations 3 and 4 have been used to measure the mean space size for each photograph taken from pre-selected locations of cells on different PDMS samples;

$S_{i}=\pi \times {R_{i}}^{2}$

(Equation 3)

$\mathrm{Mean Space}=\frac{1}{K}\sum_{i=1}^{K}S_{i}$

(Equation 4)

Where R_i_ is the radius and Si is the surface area of the i^th^ circle (in pixel) and is the number of circles in each image. 

*Statistical analysis*

To compare the results, analysis of variance (one-way ANOVA by Graph Pad Software, USA) was performed. The significant difference was reported in the cases in which the *p*-value was <0.05.

## Results and Discussion

*Bulk stiffness of substrates*

Cancer is known as a worldwide disease that has a high mortality rate. So the most important research topics belong to cancer diagnosis, treatment, and cell cancerous behavior (9-12). Like previous reports, it seems that the extracellular matrix (ECM) features such as mechanical properties (stiffness) can modulate cell phenotype, differentiation, and migration (13). Beside stiffness, surface roughness (topography) is another factor that may make an appropriate interface to create diverse cellular responses (14). Therefore, making a suitable microenvironment to eliminate the cancer cell line with physical cues probably will create a hopeful insight. 

Nowadays, in the field of cell function control and determination of its fate, the role of biomaterials is undeniable (15). In this aspect, PDMS with unique properties including transparency and the ability to change its characteristics has gained more attention in various cell-biomaterial interactions (16). Although the wide range of researches illustrates the cell response to different physical factors, this question remains as whether A549 cells as a model of lung cancer cells have different survival levels and morphological features in interaction with stiffness and surface roughness. To discuss this matter, PDMS substrate with varied stiffness and roughness were prepared and the cell behavior was studied. 

**Figure 1:**
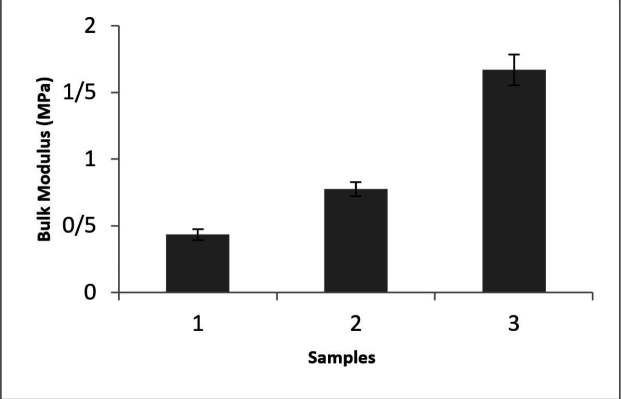
The stiffness of samples with different base: curing agent ratios (S1.2.5:1, S2. 5:1 and S3.10:1).

As stiffness can be introduced by the bulk modulus, the tensile test was performed and the bulk elastic modulus of specimens was shown in Figure 1. This spectrum represents that the highest modulus was accomplished in S3 with the proportion of 10:1 (base: curing agent). It is obvious that by adjusting the amount of base: curing agent ratio different stiffness can be obtained. To address the relationship between the A549 cell reaction on different stiffness, a conventional tensile test was considered for bulk modulus examination (Yong^’^s modulus). According to previous reports in different network density adjusted by curing agent amount various sample stiffness can be obtained (17).

*Roughness study*

**Figure 2:**
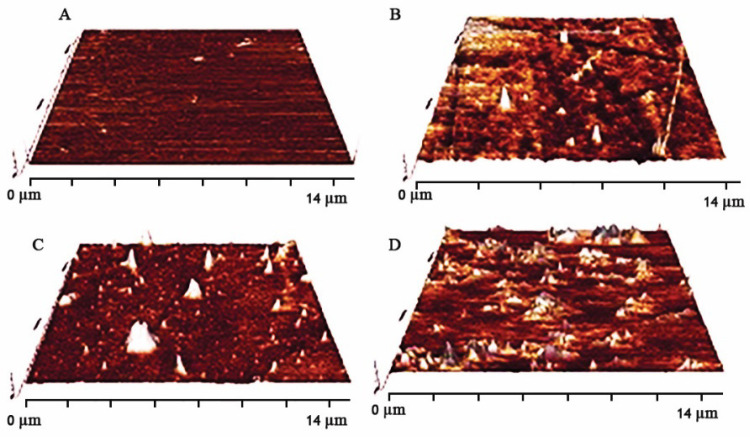
AFM images of different surface roughness: (A) control (without any plasma exposure), (B) time of exposure: 30 s, (C) time of exposure: 90 s, (D) time of exposure: 180 s

The surface roughness of substitute is one of the main characteristics which can have an effective role in a wide range of cell function, especially attachment. Atomic force microscopy (AFM) images of various surface substrates, in which exposure plasma time has been altered are shown in Figure 2. Different levels of surface roughness in PDMS samples were gained by controlling the plasma time exposure. Quantitative root mean square roughness of R2, R3, and R4 were assigned as 174.92, 189.22, and 326.7 nm, respectively. Surface topography has been altered with changes in plasma radiation time on the surface. Surface topography (roughness) was detected by atomic force microscopy (AFM) images. Ablation ability of plasma in contact with surface (PDMS substrate surface) can make different roughness. According to AFM images with an increase in time exposure, the ablation amount was enhanced hence surface roughness increased (Maximum surface roughness is referred to R4). 

*Effect of PDMS stiffness on Cell Viability*

**Figure 3:**
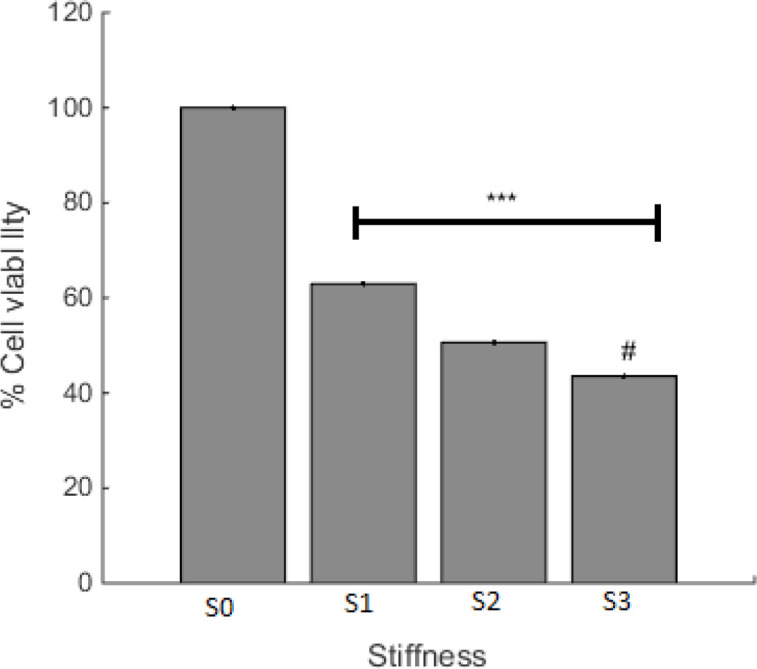
A549 Cells viability on the PDMS surfaces with different stiffness after 22 h incubation at 37 °C. S0 is the control (culture plate) and the composition and properties of S1 to S3 are presented in the methods section. ^***^p < 0.001 compared with S0. ^#^*p* < 0.05 compared with S1

Cell viability on prepared surfaces with various stiffness was investigated and the result is shown in Figure 3. The least level of cell viability was observed on the S3 surface with the minimum amount of stiffness. This finding presents a negative relationship between the degree of stiffness and cell survival for A549 cells. As cancer cell behavior is the subject of this paper, less cell viability can be desired. Accordingly, less cell viability was observed in S3 with the highest level of rigidity. It looks like that even though in the ratio of 10, base and curing agent are in the appropriate balance (based on company report) the cells tend to die and it can be related to the effect of stiffness on cell reaction. 

*Effect of PDMS roughness on cell viability*

**Figure 4:**
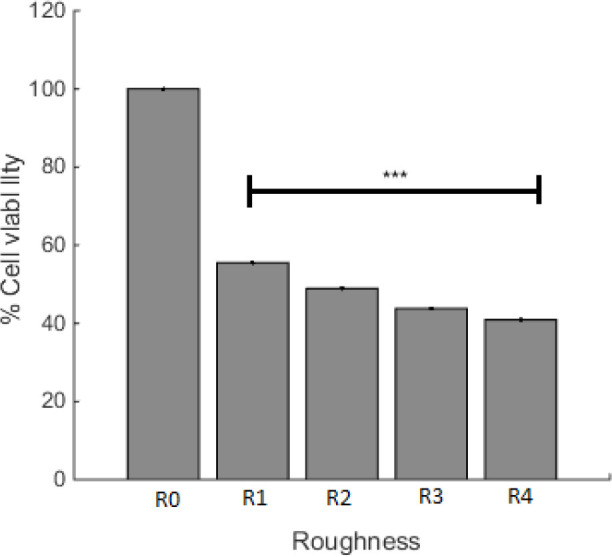
Cell viability on the PDMS with different roughness after 22 h incubation at 37 °C. R0 and R1 are cell culture and PDMS controls, respectively. R2 to R4 has increased roughness as described in the method section. ^***^p < 0.001 compared to R0

The effect of surface roughness on cell viability was studied and the result is presented in Figure 4. For the purpose of this experiment, the incubation time of A549 cells was assigned as 22 h, with R0 and R1 that are controls for plasma irradiated cell culture plate and PDMS, respectively. R2, R3, and R4 are described in the method section. Figure 4 depicts that the cell viability of different surface topography was not the same and had decreased with the increase in the PDMS roughness. Considering that the minimum viable of A549 cell in S3 was accomplished, the effect of surface roughness as an independent agent was studied in this sample and the plasma time on this sample was changed. With alteration in surface roughness, the tendency of cells to live has changed and Figure 4 depicts that the maximum level of viable cells was on the smoother surface (less roughness). 

*Cell surface calculation*

**Figure 5:**
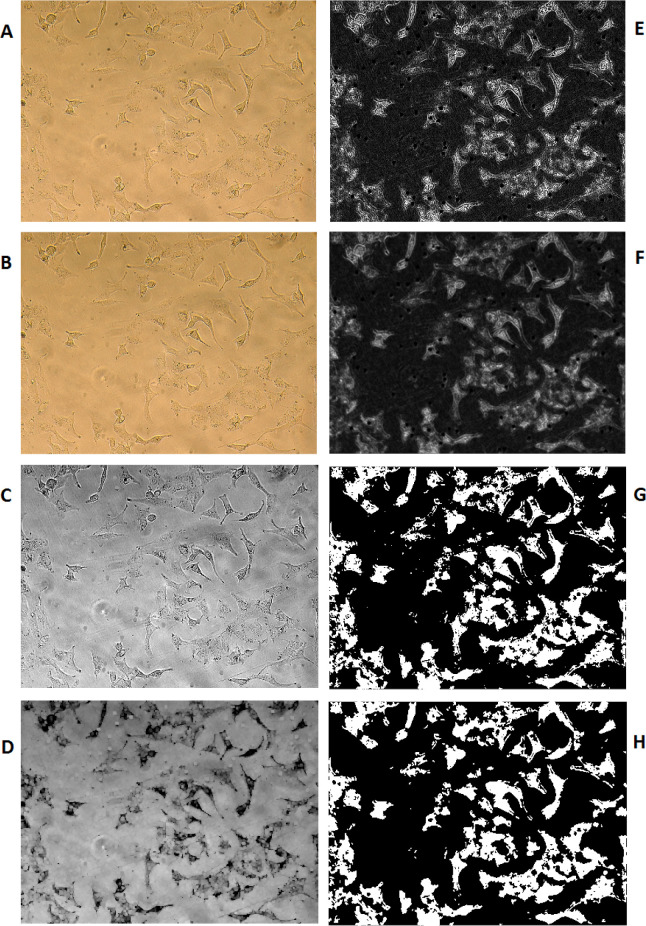
Image processing steps; (A) Original Image. (B) Original Image after reducing lens effects (C) Grayscale Image (D) Estimated background (E) Image after background removal (F) Filtered Image (G) Binary Image (H) Binary Image after eliminating noisy pixels

Figure 5 is representing the result of methods we have used to discriminate cellular surface covered by cells versus the media background; the picture is presenting an example of microscopic images that have been taken from A549 cells grown on one of the produced PDMS samples. B to H are representing the result of different processing and conversions applied by the software to count the surface area covered by cells on these PDMS samples, as explained in the method section and identified in the figure caption. In addition to cell viability, image processing was another means to the A549 cell study. The picture is showing an example of microscopic images that have been taken from A549 cells grown on one of the produced PDMS samples. B to H are representing the result of different processing and conversions applied by the software to count the surface area, covered by cells on these PDMS samples, as explained in the method section and identified in the figure caption. 

*Percentages of attached and detached cells*

**Figure 6:**
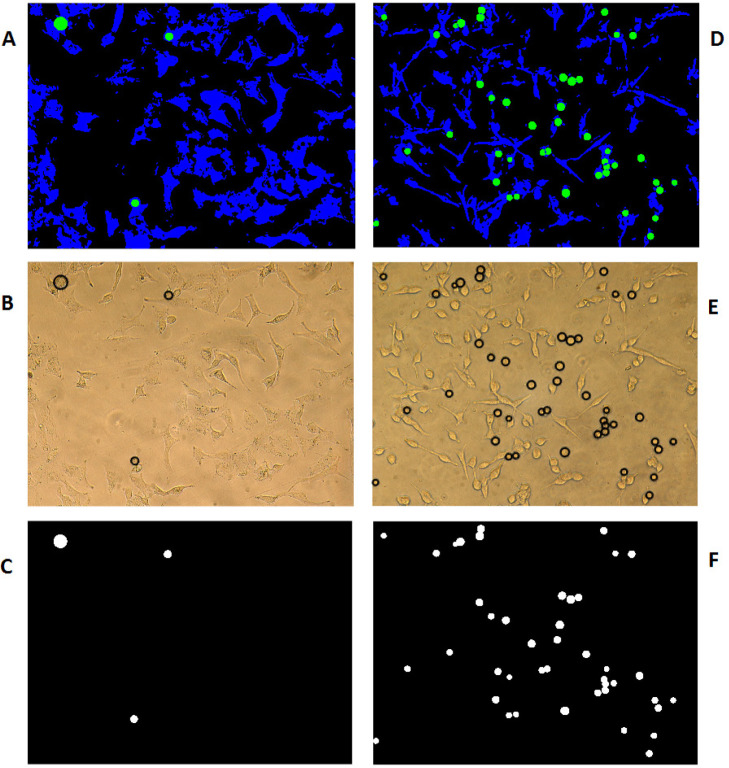
Discrimination of detached from attached cells growing on two different PDMS samples of R0 (A-C) and S2 (D-F). (A and D) Combined all categories (blue: attached cells, green: detached cells and black: medium culture). (B and E) Using the pattern originated in the previous section on the original Images to identify detached cells in the form of proportional black circles. (C and F) Display the number and size of different circles in the selected photography area

As examples for visualization of image processing codes for the determination of the attached and circled detached cells are introduced for two various PDMS produced surfaces of R0 and S2 in Figures 6A and 6D, respectively. As is shown in this figure, after the identification of an area with detached cells, the software will determine the circular detached cells and color them in black (Figures 6B and 6E). The software will then differentiate white circled detached cells from the rest of the black surface as is shown in Figures 2C and 6F. The percentage of the detached to the attached cells is calculated accordingly as described in the method section.

*Effects of surface stiffness and roughness*

**Figure 7:**
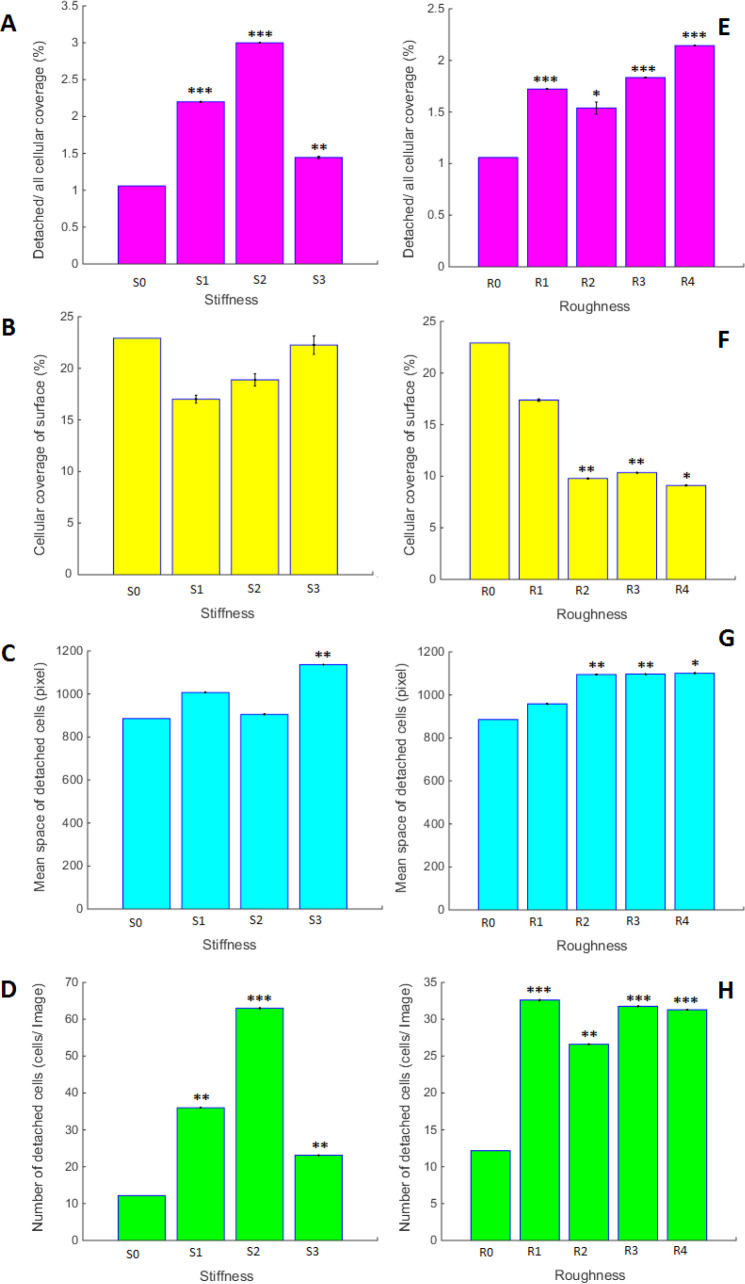
Bar graph for the comparison of A549 cells growth characteristics on PDMS surfaces with variable stiffness and roughness’s; Stars on the bars are representing the *p*-value of *t*-test for each sample compared to the control and is ^*** ^with the *p* < 0.001, ^** ^for *p* < 0. 01 and ^* ^for *p* < 0.05. Variables are defined in the method section and pictures are divided as follows; (A and E) Cellular coverage of detached cells into the entire cellular area from Images. (B and F) Cellular coverage of surfaces from Images. (C and G) Space of detached cells from Images. (D and H) Number of detached cells

Figure 7 is representing the effect of different PDMS surfaces prepared with variable stiffness and roughness on the quantity and quality of A549 cells growth. As is seen in this figure, any modification in PDMS stiffness and roughness may result in different growth characteristics of A549 cells, being statistically different from the others. All of the measured parameters are changing in a dose-response manner. The highest level of cellular coverage and mean space of detached cells is related to S3 with the maximum stiffness. The maximum number of the detached cells belongs to the samples with most rigidity. Even though high cell mortality in S3 was observed, the maximum number of the detached cells is not related to this sample. Also, roughness might be considered as the independent variable that promotes specific cellular behavior and had a regular manner in response to variable difference. As is shown in Figure 7, image processing of different variables in PDMS substrates could make individual properties of A549 cell growth characteristics. In this aspect, the substrate with more rigidity had the minimum number of detached cells, and also maximum mean space of the detached cells. Also, roughness might be considered as the independent variables that promote specific cellular behavior. Although surface with less roughness has shown the largest number of live cells, the number of detached cells was not changed in substrates with different roughness. Cellular coverage had a decreasing trend with increasing surface roughness. Our results in this section prove that even though substrates with high rigidity and roughness cause ling cancerous cell death, the cell growth features are completely different. It is worth to mention that these physical cues can promote enable signaling pathway. 

Obtained results in this research showed that the physical properties of surface substrates can influence A549 cell viability. Cellular coverage of surface, number of detached cells, means space of detached cells and detachment of cellular coverage are some of the cell growth parameters that can be modulated with surface factors. These results demonstrate that although some physical parameters on the surface can alter cell survival, these factors can have an independent effect on A549 cell growth parameters. 

## Conclusion

 In this study, PDMS substrates with different stiffness and surface roughness were prepared. Lung cancer cell survival and growth on these samples were studied and it seems that alteration in surface parameters can affect cell function independently. The results presented in this article might provide a solid base for the idea to use different physical means to manipulate the lung environment that eliminates tumor cells.
